# Frequency of Stroke and Factors Associated With It Among Old Age Hypertensive Patients in Karachi, Pakistan: A Cross-Sectional Study

**DOI:** 10.7759/cureus.23123

**Published:** 2022-03-13

**Authors:** Chamithra D Rupasinghe, Syed Ammar Bokhari, Irfan Lutfi, Maria Noureen, Fareeda Islam, Musharaf Khan, Faiqa Amin, Fares Mohammed Saeed Muthanna

**Affiliations:** 1 Internal Medicine, Manipal College of Medical Sciences, Pokhara, NPL; 2 Internal Medicine, Shifa International Hospital Islamabad, Islamabad, PAK; 3 Interventional Radiology, Shaheed Mohtarma Benazir Bhutto Medical College, Dow University of Health Sciences, Karachi, PAK; 4 Internal Medicine, Rawalpindi Medical University, Rawalpindi, PAK; 5 Medicine, Aga Khan University Hospital, Karachi, PAK; 6 Neurosurgery, Combined Military Hospital, Rawalpindi, PAK; 7 Internal Medicine, Quaid-e-Azam Medical College, Bahawalpur, PAK; 8 Pharmaceutical Care, Walailak University, Nakhon Si Thammarat, THA

**Keywords:** pakistan, prevalence, hypertension, factors, stroke

## Abstract

Introduction: Worldwide, stroke has become the major cause of mortality and morbidity among the old age population. Hypertension is one of the factors associated with stroke. Individuals with hypertension are at high risk of developing stroke. This study was conducted to determine the frequency of stroke factors associated with it among old-age hypertensive patients in Karachi, Pakistan.

Methods: It was a cross-sectional study conducted in outpatient departments (OPD) of two tertiary care hospitals of Karachi, Pakistan, including Jinnah Postgraduate Medical Center (JPMC) and Ziauddin Hospital. Eligible patients were invited to be a part of the study, and informed consent was taken from them before data collection.

Results: Multivariable logistic regression analysis showed that age (adjusted odds ratio [AOR]: 1.06, 95% CI: 1.03-1.11), smoking (AOR: 1.76, 95% CI: 1.14-2.72), lack of physical activity (AOR:2.57, 95% CI: 1.60-4.14), medication adherence (AOR: 4.22, 95% CI: 2.69-6.62), and dyslipidemia (AOR: 1.98, 95% CI: 1.23-3.21) were significantly related to prevalence of stroke in hypertensive population over 60 years or above.

Conclusion: The prevalence of stroke was high in the hypertensive population aged above 60 years and above. The study found that factors significantly associated with stroke among the hypertensive population aged 60 years or above, included age, smoking, lack of physical activity, medication adherence, BMI, and dyslipidemia.

## Introduction

Worldwide, stroke has become the major cause of mortality and morbidity among the old-age population. Stroke is defined as a disease in which the major arteries of the brain get blocked, and oxygen supply is depleted, resulting in death or disability [1]. Stroke is of several types, including Ischemic stroke, hemorrhagic stroke, and transient ischemic attack (TIA). Among them, the most common type is hemorrhagic stroke [2]. In low- and middle-income countries, the incidence of stroke has been increased in the last few years [3]. However, the incidence of stroke has been reduced significantly in developed countries. Stroke claimed the lives of 6.5 million people in 2013, resulting in 113 million disability-adjusted life years. 75.2% of all stroke deaths and 81.0% of all stroke-related disability-adjusted life years occur in developing nations [4].

Hypertension is one of the factors associated with stroke [5]. Individuals with hypertension are at high risk of developing stroke. The fundamental reason for illness and death in patients suffering from hypertension is stroke [6]. Hypertension is a modifiable risk of Stroke; therefore, it is essential to identify the confounding factors of stroke and apply the intervention [5]. However, the influencing factors of stroke in hypertensive individuals are inconsistent between research, which may be connected to geographical area and race. The Ethiopian health sector development initiative aims to reduce premature mortality from no communicable diseases (NCDs) by 12.5% between 2015/16 and 2019/20 [7]. Stroke is currently one of Ethiopia's most serious public health issues, accounting for 7% of deaths. According to a study conducted in Ethiopia, for patients admitted to intensive care units, stroke was the primary cause of death (17%) and the third most usual reason for admission of the patient in an intensive care unit (ICU) (15.2%) [8].

The burden of stroke in Asian countries is high in comparison to developed countries because of low-quality management against hypertension [9]. Pakistan is suffering from a higher prevalence of stroke because of risk factors including obesity, hypertension, and lifestyle. One-third of people who suffer a stroke develop post-stroke cognitive impairment [10]. Consequently, it is important to treat stroke properly and effectively by increasing the knowledge and early diagnosis of hypertension [3].

In addition, few studies have been conducted in Pakistan that determined multiple factors associated with stroke among old-age people with hypertension. Thus, this study was conducted to determine the prevalence of stroke among hypertension patients and factors affecting its incidence among old-age people in Karachi, Pakistan. It would help in the development of more precise interventions at a community level that is important to decrease the risk of stroke among high-risk people.

## Materials and methods

A cross-sectional study was conducted in outpatient departments (OPD) of two tertiary care hospitals in Karachi, Pakistan, including Jinnah Postgraduate Medical Center (JPMC) and Ziauddin Hospital. Eligible patients were invited to participate in the study, and informed consent was taken from them before data collection. Ethical approval for this study was taken from the Institutional Review Board (IRB) of Ziauddin University and Hospital (IRB Number: ZU_2021_04_05).

Sample size and sampling technique

The sample size was calculated using Epi Info Version 7 using the following assumptions: prevalence of stroke among old age patients with stroke is 10.8% [11], the precision of 3%, power of 80%, and non-response rate of 20%. The total sample calculated for this study was 520. Participants were recruited using a non-probability consecutive sampling technique. Patients aged 60 years or more, having hypertension, and visiting OPD for the follow-up were interviewed and assessed for the eligibility criteria. The inclusion criteria are as follows: 1) diagnosed as hypertensive patients from at least two years, 2) having an age of 60 years or more, and 3) patients who developed stroke at the age of 60 years or more. Patients with impaired mental status were excluded from the study. Moreover, patients with cancer were also excluded from the study.

Data collection procedure

Data were collected using a standard questionnaire that included sociodemographic characteristics of participants, medical history, family history of stroke, medication adherence, and behavioral factors. Sociodemographic factors included age, gender, employment status, educational status, marital status. Medical history, including the presence of stroke, family history of stroke, comorbidity other than hypertension, and body mass index (BMI), were also assessed.

A calibrated weighing scale was used to measure weight in light clothing and without shoes. Weight was measured in Kilograms (kg) with a precision of 0.01 kg. The height was measured with a stadiometer in centimeters (cm) in an upright stance with a precision of 0.1cm and without shoes. BMI was calculated by dividing weight (kg) and square of height (cm). 

Behavioral factors assessed in the current study included smoking status since the diagnosis of hypertension, physical exercise, and medication adherence towards antihypertensive medicines since the diagnosis of hypertension.

Regular physical exercise was assessed using CHNS reference [11]. Regular physical exercise is defined as more than three times a week, moderate-intensity, or more than 30 minutes each time, or participation in moderate and severe physical labor; otherwise, it is defined as a lack of exercise. Lack of exercise was categorized into two groups that are yes or no.

Medication adherence was assessed using Morisky medication adherence score to antihypertensive medicines. This tool is composed of eight questions. Each question was answered as to whether Yes (1) or No (0) [12]. A score of 7 to 8 was categorized as adherent, while a score of less than seven was categorized as non-adherent. Smoking status was categorized as smokers and non-smokers. Smokers are defined as people who have smoked continuously or cumulatively for six months or more in their life after the diagnosis of hypertension. 

A 5 mL blood sample was taken from each participant to check total cholesterol level. The following requirements must be fulfilled for dyslipidemia: a) Previous medical conditions (confirmed from the patient's previous reports or hospital data). (b) The current survey shows cholesterol of 200 mg/dL or more [13]. 

Data analysis plan

Data were analyzed using STATA version 16.0. Descriptive statistics were presented as the mean and standard deviation for continuous variables, while frequencies and percentages were calculated for categorical variables. All characteristics between participants with and without stroke were compared using an independent t-test for continuous variables and a chi-square test for categorical variables. A multivariable logistic regression model was used to estimate the adjusted odds ratio (AOR) and 95% confidence intervals (CI) of factors associated with stroke among old age patients with hypertension. P-value < 0.05 was considered statistically significant.

## Results

A total of 520 participants was included in the study. The sociodemographic and clinical characteristics of participants are shown in Table 1. The mean age of the study population is 67.36 +/- 5.84 years. The majority of study participants were males (58.27%), married (79.42%), illiterate (28.27%), and unemployed (43.27%). Moreover, third-quarter of participants had comorbidity other than hypertension (74.65%). The prevalence of stroke among people aged 60 years or more is 13.84%.

**Table 1:** Sociodemodraphic characteristics of participants Mean (Standard deviation)

Variables	Categories	n (%)
Age*		67.36 +/- 5.84
Gender	Male	303 (58.27)
	Female	217 (41.73)
Marital Status	Married	413 (79.42)
	Unmarried/widowed/separated	107 (20.58)
Education Status	No formal education	147 (28.27)
	Primary	128 (24.62)
	Secondary	84 (16.15)
	Intermediate	70 (13.46)
	Graduation	91 (17.50)
Employment Status	Unemployed	295 (56.73)
	Employed	225 (43.27)
BMI	Underweight/normal	310 (59.62)
	Overweight	124 (23.85)
	Obese	86 (16.54)
Any other comorbidity	No	128 (25.35)
	Yes	377 (74.65)

Table 2 compared the sociodemographic characteristics of participants with stroke and without stroke. Compared with people without stroke, the stroke patients were older (65.91 vs. 68.72 years), had lower education, and were unemployed (52.45% vs. 69.44%). Furthermore, gender and marital status were not significantly associated with stroke (p-value>0.05).

**Table 2:** Comparison of sociodemographic characteristics between participants with stroke and without stroke * Significant at p-value<0.05 ^Mean (standard deviation)

Variable	Categories	Stroke		P-value
		No	Yes	
Age^		65.91 +/- 5.35	68.72 +/- 6.97)	0.002*
Gender	Male	256 (57.14)	45 (62.50)	0.321
	Female	192 (42.86)	27 (37.50)	
Marital Status	Married	360 (80.36)	57 (79.15)	0.385
	Unmarried/widowed/seprated	88 (19.64)	15 (20.83)	
Education Status	No formal education	116 (25.89)	27 (37.50)	0.001*
	Primary	92 (20.53)	23 (31.94)	
	Secondary	79 (17.63)	8 (11.11)	
	Intermediate	70 (15.62)	6 (8.33)	
	Graduation	91 (20.31)	8 (10.11)	
Employment Status	Unemployed	235 (52.45)	50 (69.44)	0.001*
	Employed	213 (47.54)	22 (30.55)	

Table 3 compared the clinical characteristics of participants with stroke and without stroke. Regarding BMI, the number of overweight and obese participants is significantly higher in the stroke group (p-value=0.047). In this study, 40.28% of patients with stroke and 25.67% of patients without stroke were smoking since the diagnosis of hypertension and a significant difference is there between the two groups (p-value=0.002). Moreover, 37.05% of patients without stroke and 70.83% of patients with stroke did not adhere to anti-hypertensive medications and the difference is significant between the two groups (p-value=0.001). Other clinical characteristics that were significantly different in two groups included duration of hypertension (p-value=0.005), lack of exercise (p-value=0.001) and dyslipidemia (p-value=0.001).

**Table 3:** Comparison of clinical characteristics between participants with stroke and without stroke * Significant at p-value<0.05

Variable	Categories	Stroke		P-value
Family history of stroke	No	377 (84.15)	57 (77.79)	0.086
	Yes	71 (15.85)	16 (22.21)	
Duration of hypertension	Less than 5 years	241 (53.79)	28 (38.89)	0.005*
	>= 5 years	207 (46.21)	44 (61.11)	
BMI	Underweight/normal	281 (62.72)	37 (51.39)	0.047
	Overweight	100 (22.32)	20 (28.78)	
	Obese	72 (16.07)	15 (20.83)	
Any other comorbidity	No	109 (24.33)	20 (27.79)	0.313
	Yes	339 (75.67)	52 (72.21)	
Smoking since the diagnosis of hypertension	No	333 (74.33)	43 (59.72)	0.002*
	Yes	115 (25.67)	29 (40.28)	
Lack of exercise	No	211 (47.09)	49 (68.05)	0.001*
	Yes	237 (52.91)	23 (30.95)	
Medication adherence	No	166 (37.05)	51 (70.83)	0.001*
	Yes	282 (62.95)	21 (29.17)	
Dyslipidimia	No	355 (79.24)	45 (62.50)	0.001*
	Yes	93 (20.76)	19 (37.50)	

Table 4 shows the results of multivariable logistic regression to determine the factors associated with stroke among patients with hypertension. Multivariable logistic regression analysis showed that age (AOR: 1.06, 95% CI: 1.03-1.11), smoking (AOR: 1.76, 95% CI: 1.14-2.72), lack of physical activity (AOR:2.57, 95% CI: 1.60-4.14), medication adherence (AOR: 4.22, 95% CI: 2.69-6.62), and dyslipidemia (AOR: 1.98, 95% CI: 1.23-3.21) were significantly related to stroke in hypertensive population over 60 years or above.

**Table 4:** Factors significantly associated with stroke in old age population with hypertension (multivariable logistic regression analysis) AOR - adjusted odds ratio

Variable	Categories	AOR	95% CI	P-value
Age		1.06	1.03-1.11	0.001
BMI	Underweight/normal	Reference		
	Overweight	2.37	1.38-4.09	0.004
	Obese	1.92	1.06-3.49	0.001
Smoking since the diagnosis of hypertension	No	Reference		0.011
	Yes	1.76	1.14-2.72	
Lack of exercise	No	Reference		0.003
	Yes	2.57	1.60-4.14	
Medication adherence	No	4.22	2.69-6.62	0.001
	Yes	Reference		
Dyslipidimia	No	Reference		0.005
	Yes	1.98	1.23-3.21	

## Discussion

In this study, the prevalence of stroke in the hypertensive population over 60 years or above is 13.84%. The prevalence of stroke was significantly associated with age, BMI, smoking, lack of physical activity, medication adherence, and dyslipidemia. Prevalence of stroke in hypertensive population over 60 years or above found in the current was remarkably similar to findings of the study conducted in China [14]. This result was approximately two to three times higher than the standardized prevalence of stroke in the general population reported by other studies [15,16].

Identification of the factors associated with stroke among hypertensive patients can aid in the prevention of the occurrence of stroke in old age people. The current study identified certain factors associated with stroke, including age, BMI, smoking, lack of physical activity, medication adherence, and dyslipidemia. Studies conducted by Owolabi et al. and Mekonen et al. found that dyslipidemia and lack of exercise are significant factors related to the prevalence of stroke in hypertensive patients [13,17].

The current study shows that the risk of stroke is increased with an increase in age. According to the Thai Stroke Registry, the average age of patients at the beginning of ischemic stroke in Thailand is around 65 years old [18]. This could be related to the biological effect of increased vascular resistance as a result of arterial thickening as people age [19].

Patients with dyslipidemia were more likely to be at a greater risk for stroke as compared to the patients without dyslipidemia. Studies conducted in Tanzania and Nigeria showed similar findings [17,20]. This could be because cholesterol directly impacts blood circulation, which can lead to a stroke. However, according to the study conducted by Ismail et al., there was no statistically significant difference in Cholesterol variation between the groups with and without stroke [21]. This is possible because of the difference in study design and sample size. The current study also showed that patients who did not adhere to prescribed drugs are more likely to develop a stroke than patients who adhered to drugs. The study conducted by Chowdhury et al. on 74 hypertensive patients who suffered from a stroke showed that 34% of patients did not adhere to antihypertensive medications [22].

Obesity and overweight are severe public health issues linked to a variety of serious medical diseases, including stroke. Weight loss is recommended by the American College of Cardiology and the American Heart Association (ACC/AHA) as a preventative measure for stroke and other cardiovascular problems [23]. The current study has also found that obese and overweight individuals are at greater risk of developing stroke. The study conducted by Cho et al. suggested that weight gain is associated with an increased incidence rate of TIA [24]. Thus, management of weight needs to be an optimal method for preventing stroke.

Because stroke is multifactorial, people, particularly those at risk, should be aware of their amount of exposure and stay away from those risk factors. Identifying present medical issues is the first step to understanding and gaining knowledge about risk factors. Therefore, health care professionals need to guide individuals with hypertension about disease management, including medication adherence, proper physical activity, and proper dietary intake. Of course, hypertension contributes to stroke events on its own; however, when other modifiable lifestyle factors (such as smoking, excessive alcohol consumption, physical inactivity or sedentary lifestyle, obesity, high cholesterol, and poor dietary habits) are poorly managed, the risk of stroke is significantly increased.

The current study has certain limitations. To begin with, the causal association between these factors and stroke cannot be determined because this is a cross-sectional study. Second, certain variables such as the condition of the carotid artery, arterial fibrillation, blood pressure control, and dietary patterns were not determined in the current study. Third, recall bias might be there among patients with stroke, as it would be difficult for them to remind about their previous health-related behaviors. Lastly, due to our failure to precisely determine the kind of stroke, we were unable to distinguish between ischemic and hemorrhagic strokes, which may have influenced our findings. Thus, in the future, larger and high-quality prospective cohort studies are required to verify and explore the factors of stroke old age patients with hypertension.

## Conclusions

The prevalence of stroke was high in the hypertensive population aged above 60 years and above. The study found that factors significantly associated with stroke among the hypertensive population aged above 60 years and above included age, BMI, smoking, lack of physical activity, medication adherence, and dyslipidemia. Considering the burden of stroke on an individual level and also on the health care system, it is important for health care professionals to develop prevention mechanisms and effective interventions to improve primary stroke prevention. Moreover, interventions should also target the identification of high-risk patients to provide them with education about the management of hypertension.
